# Case of Poisoning by Opium, in Which Electro-Magnetism Was Employed

**Published:** 1846-11

**Authors:** J. J. Colahan


*Case of Poisoning by Opium, in which Electro-Magnetism was Employed.* By J. J. Colahan, Esq.—An infant of perfect conformation had been given 25 drops of laudanum to procure sleep. This was at one o'clock, p. m.; at eight o'clock, Mr. Colahan saw the child; the respiration was hurried, occasionally there was stridor, with other signs of acute bronchial irritation; the pulse rapid, but distinct; the physical symptoms, Mr. Colahan remarks, constituted a group exactly resembling laryngitis. An emetic was ordered; in eight hours' time profound torpor and sleep came on, when another emetic was given, and means adapted to rouse the child.

Tartar emetic solution was given every ten minutes in half grain doses, in order to relieve the lungs of the mucus. In spite of every effort, the vital energy began to decrease, and Mr. C. called in Dr. Barry, surgeon to the Maternity Hospital, who suggested the use of galvanism. It was immediately had recourse to.

The strength of the galvanic current was at first comparatively feeble; the shock caused by its influence, although perceptible, was slight, and proportionally diminished after application to the different most sensitive parts of the body. The strength was now increased by sulphuric acid, and a rapid current of electricity developed, to the powerful stimulus of which the infant was exposed, alternately applied to different parts. The physioligical effect was now apparent, and it was not until the regulator was moved to its highest point, and the flow of electricity had reached its maximum intensity, that complete signs of revival were produced, causing involuntary muscular contractions, with frequent voluntary efforts of the child to get released from its painful position. The galvanic influence was not deemed prudent to be discontinued for five hours, at the close of which sensibility was evident and complete, with restoration of the functions of the nervous centres. Gradual and progressive recovery continued, and in a few days the infant was perfectly convalescent.

*Remarks.*—The quantity of laudanum given in this case I ascertained with tolerable certainty (from an examination of the portion that remained) to have been at least twenty-five minims, which was corroborated by Dr. Barry, who obtained separate pennyworths, procured by different persons, and his calculation tended to a similar result. From the enormous dose relative to the child's age, and recovery from its effects, a weak preparation was supposed to have been administered. Subsequent to the reading of the paper a portion of laudanum obtained from the same stock, which had been made for some time previous, was procured by Dr. Barry, which was subjected to Dr. Christison's careful analysis, and proved to have been of regular strength.—*Dublin Medical Press.—Braithwaite's Ret.*

